# CCL: an algorithm for the efficient comparison of clusters

**DOI:** 10.1107/S0021889813006894

**Published:** 2013-04-06

**Authors:** R. Hundt, J. C. Schön, S. Neelamraju, J. Zagorac, M. Jansen

**Affiliations:** a Institut für Anorganische Chemie, Universität Bonn, Gerhard-Domagk-Strasse 1, D-53121 Bonn, Germany; b Max-Planck-Institut für Festkörperforschung, Heisenbergstrasse 1, D-70569 Stuttgart, Germany

**Keywords:** cluster structures, structure prediction, structure determination, nanomaterials, computer programs

## Abstract

The systematic comparison of the atomic structure of solids and clusters has become an important task in crystallography, chemistry, physics and materials science, in particular in the context of structure prediction and structure determination of nanomaterials. In this work, an efficient and robust algorithm for the comparison of cluster structures is presented, which is based on the mapping of the point patterns of the two clusters onto each other. This algorithm has been implemented as the module CCL in the structure visualization and analysis program *KPLOT*.

## Abstract 2

An efficient and robust algorithm for the comparison of clusters is presented. Several illustrative example applications are given, including the screening of sets of clusters generated during global optimizations and Monte Carlo/molecular dymanics simulations, the identification of specific structure fragments inside large clusters, and the study of structure–substructure relations of periodic crystals.

## 1. Introduction   

The degree of similarity between two given cluster structures is an important aspect of many investigations in crystallography, chemistry, physics and materials science, in particular in the context of structure prediction and structure determination of nanomaterials. Applications range from the unique classification of clusters and their embedding in crystalline structures (Driess & Nöth, 2005 ▶), through the study of phase transitions, to the ubiquitous task of analyzing newly discovered clusters *via* comparisons of their structure with those of known clusters exhibiting similar structural characteristics. More recently, the field of computer-assisted structure prediction and structure determination of clusters (Wales *et al.*, 2000 ▶; Ferrando *et al.*, 2008 ▶) or even of proteins/polymers (Wales *et al.*, 2000 ▶; Schön & Jansen, 2001*a* ▶,*b* ▶) has resulted in procedures that generate thousands of so-called structure candidates (Schön & Jansen, 1996 ▶, 2001*a* ▶,*b* ▶; Sokol *et al.*, 2010 ▶). These need to be sorted in some automatic way, in order to eliminate large numbers of duplicates that would otherwise clog up the refinement stage of the various search algorithms. While programs that allow the automated determination of, for example, the symmetries (Pilati & Forni, 1998 ▶) and connectivity (Blatov, 2006 ▶) of a simulated cluster structure can constitute a first step in this direction, more comprehensive procedures are clearly needed.

The theoretical and algorithmic work aimed at developing reliable and useful comparison procedures tends to deal with one or several of three different aspects of the overall problem: the unique classification from a crystallographic or chemical point of view, the development of meaningful ‘figures of merit’ that yield a quantitative measure of the degree of similarity between two clusters, and finally the construction of algorithms that perform an unbiased automatic structure comparison. Here one should note that such algorithms could take very different shapes depending on whether the goal is the determination of the quantitative similarity between two structures already known to be related, or whether basic similarity between a new structure and one drawn from the database of known cluster structures needs to be established.

In the field of crystalline structures, one class of measures of similarity are based on distance differences between atoms belonging to related structures (Nishikawa *et al.*, 1972 ▶), leading to error-scaled difference distance matrices (Schneider, 2000 ▶), or employ distance–distance scatter plots based on the shortest contacts for all atoms in both structures (Dziubek & Katrusiak, 2004 ▶). Such measures are probably most useful for analyzing the small changes in structures due to external influences such as changes of temperature and pressure, and could be applied to analogous cluster studies. Similar atom–atom or fragment–fragment distances are also employed in the classification schemes proposed by Chisholm & Motherwell (2005 ▶) and Willighagen *et al.* (2005 ▶).

In these procedures, the atom–atom distances of what constitutes essentially a large cluster cut out of the periodic crystal structure (or out of the interior of a macroscopically large cluster representing a nanocrystal) are computed, resulting in a direct-space approximation of the radial distribution function (RDF) of the crystal or cluster. The difference between the RDFs of the two structures under consideration is then employed as the figure of merit, where additional weight factors can be introduced based on ‘chemically and/or energetically important’ atom–atom distances (Willighagen *et al.*, 2005 ▶). Clearly, this approach *via* evaluation of the RDF can be (and has been) applied to the classification and comparison of small clusters. We note that it is implicitly assumed that the RDF will ensure a clear decision on whether two clusters or crystal structures are equal. However, this is not true in general – a straightforward counterexample is two enantiomorphic structures.

As a popular alternative, in the field of structure prediction of clusters, one often relies on the energy of the relaxed cluster to serve as a proxy for the classification of cluster structures. This criterion appears to work quite nicely if all structures have been carefully minimized with a high-quality local optimizer. However, we again encounter a number of issues that make this approach problematic, both in principle and in practice. For one, different methods used to compute the energy of the clusters typically give different results, and thus using energy as a criterion beyond as an initial screening tool is inherently questionable. For example, the investigation of the space of feasible clusters might have been performed (by different research groups) using different energy functions, *e.g.* the Hartree–Fock and density functional theory approaches, and the additional local minimization required for an energy-based comparison could prove to be rather expensive computationally. Closely connected is the observation that the precision of the energy calculations employed is limited, in particular regarding the location of the energy minimum when performing the minimization; it may well be that the minimization routine will stop at slightly different locations inside the minimum basin, with slightly different energies, especially if stochastic quenches and/or *ab initio* energy functions are employed. This can lead to conflation of clusters that are structurally different but have nearly the ‘same’ energy. This can easily happen in alloy-like intermetallic clusters where homotops can be present, which differ only very slightly by energy but exhibit a different distribution of the atoms over the underlying set of atom positions, *i.e.* they exhibit a different chemical order. Conversely, one might be interested in finding out which of the many energetically slightly different cluster minima exhibit the same structure if one treats all atoms as being of the same type, *i.e.* one would like to classify the clusters found according to whether they are (*a*) different with regard to the underlying atom arrangement, (*b*) homotops or (*c*) really the same structure.

Another classical problem case are enantiomorphic clusters, which have identical energies but whose structures are mirror images (Pacheco-Contreras *et al.*, 2012 ▶). This is not only an academic issue; such structure pairs are of special interest because helical clusters are expected to be of relevance in chiral catalysis (Szöllosi *et al.*, 2005 ▶) and nanoscale devices (Chakrabarti & Wales, 2011 ▶). We note that, as mentioned above, the problem of enantiomorphic clusters cannot be addressed using the radial distribution function of the cluster, either. Finally, we want to be able to compare clusters that have been generated for different chemical systems. Here, a classification by comparing energies is obviously not possible, and even a comparison using scaled atomic distances in the radial distribution function can lead to difficulties.

In contrast, the approach presented in this work is based on mapping the two point patterns, *i.e.* the positions of the centers of mass of the atoms in a cluster, onto each other in direct space without first performing any kind of standardization except an (optional) volume rescaling. This robust procedure takes possible structural distortions fully into account, while also allowing us to treat various special and/or interesting cases such as enantiomeric structures, alloy structures or partial structural agreement between the structures. After a description of the algorithm that has been implemented as the module CCL in our structure visualization and analysis program *KPLOT* (Hundt, 2011 ▶), we present a number of examples where the procedure has been applied, and close with a discussion of its merits and limitations.

## 2. Comparison of two point patterns   

### 2.1. General procedure   

The general concept behind our approach is partly inspired by our algorithm for the comparison of two periodic structures, CMPZ (Hundt *et al.*, 2006 ▶). We have to find a special inhomogeneous orthogonal transformation 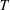, *i.e.* a map consisting only of shifts and rotations of the cluster, that maps cluster structure A to cluster structure B such that every atom belonging to structure A is mapped onto a unique atom in structure B, and that the inverse transformation 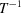 maps every atom belonging to B onto a unique atom in structure A. We note that this method can also be used to show whether a particular cluster A can be embedded into a larger cluster B or even into a periodic crystalline structure C.

### 2.2. Algorithmic implementation   

The main algorithmic tasks that need to be addressed are threefold:

(1) Map two clusters belonging to the same system onto each other, and provide a quantitative measure of their similarity

(2) Compare two clusters containing the same number of atoms but belonging to different chemical systems

(3) Check, whether a given cluster can be embedded inside a larger cluster or a periodic crystal

In practice, the comparison algorithm follows the following strategy for checking whether such a mapping exists:

(1) When dealing with two clusters generated for the same chemical system, it is obviously not necessary to rescale the clusters. However, if different types of atoms are involved, a geometrical mapping only makes sense if the atomic distances between neighboring atoms are the same. This can be achieved by rescaling the clusters, either automatically or explicitly by hand. To perform an automatic rescaling, we first check whether both clusters contain the same number of atoms, and then compute the volume of a convex hull around each cluster. To achieve this, we determine the ellipsoids that approximate the two structures (*via* their moment of inertia matrix), compute the ellipsoids’ volumes and perform an overall rescaling of the clusters such that the ellipsoids have the same volume. If the two clusters contain different numbers of atoms, one has to follow procedure UFRA (see below), in order to check for the existence of an embedding of the smaller cluster into the larger one.

(2) Next, a list of candidates for the transformation 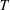 is generated, which fulfill the condition that they map the atoms belonging to a three-atom frame in structure A to a three-atom frame in structure B. We generate such test frames in cluster A, either automatically or by hand, by selecting triplets of atoms that form the corners of a maximum size triangle. Here, we make sure that the vectors connecting these atoms do not form any angle smaller than 20°.^1^ This frame is mapped to structure B by rotation and translation in such a way that the three atoms in A are each mapped to an atom in B. Of course, these three target atoms must be of the same type (called the ‘reference atom’ type).^2^ We then repeat this procedure for all possible further maps of the frame to structure B, in order to find alternative candidates for the transformation 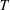.

(3) We next check whether any of the transformations 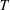 will map the atom content of cluster A to cluster B, within the tolerance 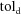. Here, 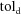 is the maximally allowed separation of the image of an atom in A from a target atom in B (in ångström). Note that, in contrast to the case of periodic structures, it is not necessary to prove that the atoms of cluster B can be mapped to cluster A: in the case of comparison of two clusters of the same size, the existence of 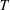 mapping cluster A uniquely to B guarantees the existence of the inverse map 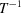 which maps cluster B to cluster A. Furthermore, if one checks whether cluster A can be mapped into structure B (a larger cluster or a periodic structure), the reverse map is not required.

As we have pointed out above, real structures, whether drawn from experiment or simulations, will always exhibit some differences. Thus, when mapping the frames for determining candidate transformations, distortions of the frame are allowed within the tolerances.

If the comparison has been successful, the required transformation matrix and the shift are printed. If the amount of misfit is of interest, one needs to employ additional tools within *KPLOT* to generate an optimal matching of the two structures for which the comparison has been successful.

These tasks can be achieved *via* the following list of commands in *KPLOT*:

(*a*) UFR

(1) Triplets defined by hand, rescaling defined by hand

(2) Possible mappings based on triplets generated

(3) Visual inspection of application of mapping to the whole cluster performed

(*b*) UFRA

(1) Triplets defined by hand, rescaling defined by hand

(2) Possible mapping based on triplets generated and applied to the whole cluster based on the given tolerances

(3) Visual inspection of application of mapping to the whole cluster performed if desired

(*c*) CCL

(1) Automated rescaling of cluster (*via* moments of inertia ellipsoids for two clusters of same size)

(2) Automated selection of handle (criterion: largest triangle of atom triplets; avoid narrow triangles)

(3) Successful mapping checked on the basis of given tolerances

(4) Visual inspection of application of mapping to the whole cluster performed if desired

(5) Options in CCL: select tolerances of mapping; set rescaling factor explicitly; perform the comparison in an atom-specific/non-specific fashion, yielding atom type mappings

We note that all the individual commands used in defining parameters, performing display of results *etc*. can be automated within *KPLOT* as a macro. Furthermore, one should keep in mind that for clusters that barely match within the given tolerances it can happen that, as a result of the scaling step involved, cluster A shows a match with cluster B, but not cluster B with cluster A. In this case, the clusters should be considered similar, nevertheless, as one will see from visual inspection.

## 3. Illustrative examples   

The following examples have been selected to give an overview of the large variety of questions in cluster and solid state science that can be successfully addressed using the structure comparison algorithms presented in the previous section.

Note that CCL is implemented within the program *KPLOT* and is executed with the single command ‘CCL’. Of course, the structures A and B have to be read into or defined in *KPLOT* first. In the examples below, we always explain what each of the *KPLOT* commands means; for details of the command syntax, we refer to the *KPLOT* manual (Hundt, 2011 ▶). Note that, if one intends to automatically compare many structures one has compiled, it is recommended to write a control script, which (*a*) reformats these structures into *KPLOT* structure files, (*b*) reads them pairwise into *KPLOT* and executes CCL with the default options, and finally (*c*) registers whether the comparison has been successful. For this purpose, the script programs *LOADCLUST* and *FILTERCLUST* have been developed, which scan through all the data files produced, for example, by the global landscape exploration package *G42* (Schön & Jansen, 1996 ▶) (*LOADCLUST*), convert them into *KPLOT*-readable files, and perform structure comparisons both among these structures (*FILTERCLUST*) and of these structures with an already prepared set of known cluster structures.

These scripts can be easily modified to include conversion to the *KPLOT* format from other file formats or other file-naming conventions. Please feel free to contact the authors for further information if necessary.

### 3.1. Example 1: comparisons of sets of clusters   

As an example, we present an analysis of results of global landscape explorations for clusters of different sizes and systems, (KMgF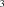)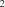, (KMgF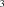)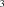 and (KZnF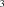)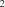 clusters.

#### (KMgF_3_)_*z*_ 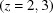   

3.1.1.

After performing simulated annealing runs on the energy landscape of the (KMgF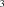)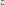 clusters with 30 and 80 stochastic quenches followed by conjugate gradient minimizations along the trajectories of the walkers for two (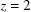) and three (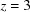) formula units, respectively, the two sets of minima were analyzed, and the number of duplicates was determined using the CCL algorithm.

In the case of (KMgF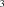)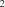, among the 30 minima found, 12 distinct minima exist. Of these, one minimum was found eight times, one four times, three three times and two two times, and the remaining five appeared only once each during the global search. Similarly, for (KMgF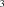)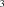, the 80 minima found can be classified into 58 distinct minima, where one appeared six times and another one four times. Furthermore, three were found three times and eight two times, and the remaining 45 were seen only once.

Since the structures had been fully optimized in careful local optimizations, we find the same classification if we employ the energies of the structures as criterion.

#### 3.1.2. (KZnF_3_)_2_   

A system where ‘similar’ structures to (KMgF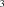)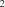 might be expected is (KZnF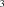)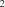. Again performing 80 quenches plus gradient minimizations along simulated annealing runs yielded 25 structurally distinct minima: one appeared eight times, five seven times, one six times, one five times, three three times and three two times, and the remaining 11 were found only once. However, if one employs energy as criterion, only 24 different minima are found. The reason is that two of the minimum structures are enantiomers, *i.e*. they are mirror images with identical energies. Using only energy as a criterion would have missed the existence of this pair of enantiomorphic structures (termed structure1 and structure2). To prove this, we inverted one of these structures using the ZG command in *KPLOT*, and then performed the cluster comparison as usual with CCL:

get structure1: load file of structure1

zg 3 12 1: invert the coordinates of all ten atoms belonging to the cluster^3^

ns: move structure1 into the background

get structure2: load file of structure2

ccl **1**1: compare the (inverted) structure1 and structure2, using the default tolerances (**) and no rescaling (1) for the default choice of atoms, *i.e.* all (**) allowing only mapping between atoms of the same type (1)

The resulting output of *KPLOT* yields the required mapping.

#### 3.1.3. (KMgF_3_)_2_ *versus* (KZnF_3_)_2_   

Next, we performed a structure comparison between the structures found for (KMgF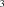)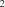 and (KZnF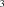)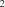. Clearly, a comparison by energy or pair distribution function is not suitable in this case. Here, we used the (default) CCL option that the clusters can be rescaled if necessary to allow a successful mapping to exist. We find that seven out of the 12 (KMgF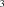)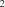 cluster isomers agree geometrically with one of the rescaled (KZnF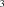)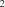 structures for 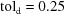 and ten out of 12 for 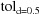.

#### 3.1.4. Clusters generated from a Monte Carlo simulation with nonzero temperature   

In the previous subsections, the structures being compared corresponded to refined local minima on the energy landscape of the chemical system(s). In a final application, we performed short constant-temperature simulations for the (KMgF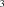)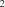 system and compared the structures seen along the trajectories.

Two temperatures were chosen, 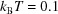
 eV and 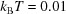
 eV (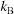 is the Boltzmann constant), and 60 and 40 structures were selected along the trajectories, respectively. In the former case, the energy of these structures was spread over an interval of 0.12 eV, and in the latter case the spread was 0.013 eV. Table 1 ▶ gives the percentage of similar structures found for the two temperatures, as function of the tolerance parameter 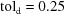 (default), 0.5, 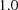 and 1.5. For the lower temperature, on average for any structure about one-quarter of all the structures are already similar geometrically for the default value of 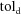, and all structures are similar for the two largest values of 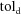. In contrast, the run at higher temperature produces essentially no pairs of similar structures for the two lower tolerances, and even at the highest tolerance, only about 16% of the structures are similar to a given structure on average. Note that, if one tries to increase this percentage by employing even higher tolerance values, it is no longer possible to find unique mappings between the clusters, since now one atom can be mapped to several target atoms within the (too) large tolerance radius.

We note that, in contrast to the comparison of well refined local minima, there is now only a weak correlation between the energies of the structures and their geometrical similarity.

### 3.2. Example 2: presence of a polyhedron inside a large cluster   

A common task one faces when analyzing the results of cluster simulations is to decide to what extent pieces of the periodic crystal structure in the chemical system are already present inside the cluster. Usually, one performs a visual inspection, but the use of CCL or UFRA provides an opportunity to automate this check. We note that, by repeating the mapping for different values of the tolerance parameter 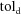 in the UFRA command giving the allowed distance mismatch of the mapping, we can gain a quantitative figure of merit: how well the cut-out from the ideal crystal matches the, frequently distorted, counterpart inside the cluster.

**Figure 1:**
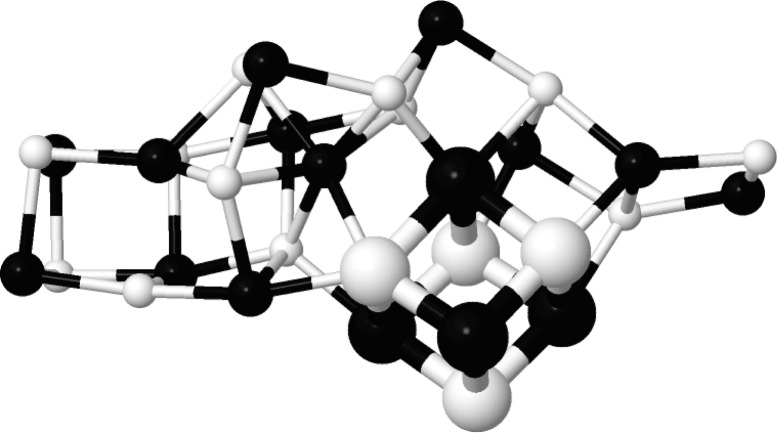
Na
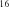
Cl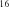 atom cluster containing an eight-atom cube-shaped cut out from the rock salt structure (depicted as large spheres). Na atoms and Cl atoms are colored white and black, respectively.

As an example, we have checked, for 3200 low-energy 32-atom NaCl clusters that were found in minimum basins during a global optimization study, whether they contain an eight-atom cube consisting of four Na and four Cl atoms cut out of the periodic NaCl rock salt structure (*cf.* Fig. 1 ▶). Using very tight tolerances (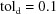) showed that only about 4% of the structures contained an essentially undistorted cube. For the default value of the allowed distortion of the mapping, about 23% of the clusters observed contained a moderately distorted cube, while allowing extremely distorted cube-like portions of the clusters (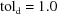) resulted in a 26% fraction (*cf.* Table 2 ▶).

Furthermore, about 80% of the clusters with a very low energy (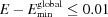
 eV atom^−1^) contained at least one such Na
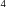
Cl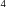 cube, a percentage that was reduced to about 15% for clusters with somewhat higher energies (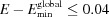
 eV atom^−1^), while the clusters corresponding to high-energy minima did not exhibit any cube-like substructure. This comparison required less than two minutes real time on a single 2.4 GHz Intel processor.

### 3.3. Example 3: existence of a substructure inside a periodic structure   

A common feature of the analysis of crystalline structures consists in finding structural relationships of newly discovered structures to already well known structures or parts of them, which can be visualized as a cluster of atoms. In particular, information about whether the new structure (or part of it) constitutes a substructure of the known one can often give valuable information about, for example, the type of chemical bonding in the compound.

As a simple illustrative example, we consider the structural relationship of the perovskite structure of CaTiO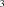 and the structure of ReO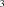 [note that this system had already been analyzed with an earlier version of UFRA (Hundt *et al.*, 2006 ▶); we present it again for pedagogical reasons]. The structural data for CaTiO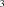 and ReO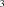 are taken from the ICSD (Bergerhoff *et al.*, 1983 ▶; ICSD-FIZ-Karlsruhe, 2005 ▶) (Nos. 31865 and 16810, respectively), and we want to check whether the ReO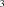 structure is contained in the CaTiO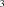 structure. For this, we first read in these two structures, where CaTiO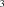 and ReO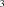 constitute structure1 (in the background) and structure2 (in the foreground), respectively. As fragment *F*, we choose the content of eight unit cells of ReO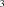, using the command

ATB 2 * * 1.0 1.0 1.0

All atoms with relative coordinates (*x*, *y*, *z*) ranging from (−1, −1, −1) to (+1, +1, +1) are selected as part of the fragment. All locations are given with respect to the original unit cell.

We select as handle (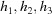) the following atoms:

*h*_1_: Re 1 355501 0.00000 0.00000 0.00000: Re atom at (0, 0, 0)

*h*_2_: O 1 455402 0.00000 0.00000 -0.50000: O atom at (0, 0, −0.5)

*h*_3_: O 1 455501 0.50000 0.00000 0.00000: O atom at (0.5, 0, 0)

The notation ‘355501’ *etc.* is commonly used to describe the location of a particular atom in a periodic structure, for example, when generating *ORTEP* plots (Johnson, 1965 ▶). The first digit refers to the number of the atom in the asymmetric unit (here No. 3), the next three digits refer to the position of the unit cell in the periodic structure where the atom is placed (here the reference unit cell), and the last two digits give the number of the symmetry operation used to generate the atom from the one in the asymmetric unit (here the identity operation). For more details, we refer to the *KPLOT* (Hundt, 2011 ▶) or *ORTEP* (Johnson, 1965 ▶) manuals.

Since the distances Ti—O (1.898 Å) and Re—O (1.867 Å) are very similar, one does not need to perform a rescaling. However, since we are trying to map a rather large fragment, one should increase the value of 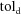. The *KPLOT* command

UFRA 355501 455402 455501 1: fit handle, and check whether F matches

then results in the output

(1) VZDP 355501 455402 455501 455501 555501 555502

VZDP is the *KPLOT* command describing the rotations and shifts of *F* that need to be executed, in order to achieve a successful mapping of *F* onto structure1. The first and last three numbers indicate the atoms describing the handle and those atoms onto which the handle has been mapped, respectively.

**Figure 2:**
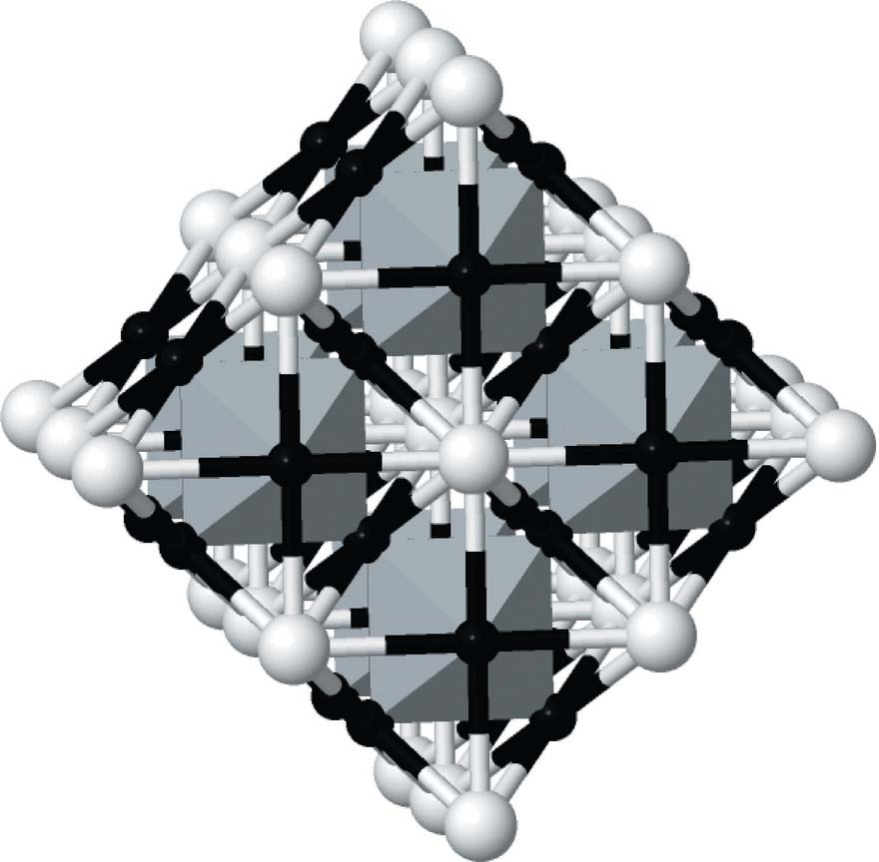
Cut out from the CaTiO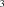 structure with the TiO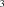 substructure, which is geometrically equivalent to the ReO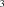 structure, highlighted as a network of TiO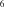 octahedra. Ca atoms and O atoms are colored white and black, respectively; Ti atoms are at the centers of the octahedra (not visible).

This output indicates that a successful positioning of the handle inside the perovskite structure has been found, such that all atoms of the fragment have been mapped to appropriate partners in the structure of CaTiO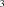. Atoms that correspond to each other can now be displayed in a plot that superimposes the fragment on the perovskite structure (*cf.* Fig. 2 ▶).

## 4. Discussion   

In the previous two sections, we have described the CCL algorithm and its implementation, together with the related UFR/UFRA algorithm, and given several typical application examples. Our experience has shown the algorithm as implemented in *KPLOT* to be quite robust and easy to use: no preparatory work is needed regarding the structures to be compared; any cell parameters, settings and symmetries can be employed in describing the clusters (and possible periodic target structures); and after entering the structure data into *KPLOT*, a few simple commands suffice to perform the comparison.

An important reason why we have implemented the algorithm as part of the program *KPLOT* is the visualization function of *KPLOT*: one can easily depict the way the two structures need to be transformed in order to match, and if no match is possible, one can use the tools available in *KPLOT* to identify the reason for the mismatch.^4^

As has been described above, the algorithm CCL is based on the comparison of two cluster structures represented as sets of points, where the identity of these structures is established by ensuring that there exists a one-to-one special inhomogeneous orthogonal map between the two sets of points, and the sum over the distances between the locations of the points in one set and the images of the points in the second set is sufficiently small. Thus, we do not automatically deal with structures that are represented by connectivity graphs based on ‘chemical connectivity’ (*e.g.* chemical bonds). If the similarity of these connectivity graphs correlates with their geometrical similarity, CCL will also recognize these structures as identical, but if no such correlation exists, CCL cannot be applied. On the other hand, CCL makes a distinction between structures that are enantiomers. If one wants to check whether two clusters are enantiomers, one can execute the ZG command in *KPLOT*, which effects an inversion on the structure for one of the test clusters before performing the comparison (*cf.* §3.1.2).

The match by our procedure essentially consists of two steps: we check possible three-atom frame matches, and then follow this by a match of the whole structures based on the map established in the first step. In principle, one could refine this process by using the information gained in the structure comparison to improve on the cluster match such that the map is ‘optimal’ not only for the handle but for the whole structure. However, our experience has shown that this does not lead to any qualitative changes regarding the identity of the two structures, and yields only very minor quantitative improvements in the agreement. Since the most common application of the CCL algorithm is the comparison of thousands of cluster structures with each other (*e.g.* when sorting the results of global search algorithms, or of Monte Carlo/molecular dynamics simulations) and with structures drawn from large databases, speed is an important criterion. Thus, we have decided not to implement such a refinement cycle as part of the CCL command. However, such a refinement is also possible within *KPLOT* using a combination of the ID and the AUF commands. For more details, we refer to the *KPLOT* manual.

Another option that has not been implemented because it would cause an excessive slow down in the performance is an automatic check for best ‘partial agreement’ of the two structures. In principle, one can perform a multiple comparison between smaller and smaller subsets of equal size drawn from the two clusters, if no successful total match between the two structures has been found. This would allow us to generate optimal incomplete matches, giving information about which parts of the two structures can be mapped onto each other. However, the amount of data produced and the time required to perform this multitude of checks is huge, and thus we have removed this option in the final implementation. For instance, take two clusters with 20 atoms each per unit cell, and attempt to find a possible match by employing subsets of 15 atoms from each structure: the number of possible structure comparisons that need to be performed would be 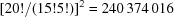. Since information about the best partial agreement is usually only of interest if one already suspects the two structures to be related, it tends to be more efficient to check their relationship by hand and/or to define a specific promising structure fragment/substructure for which an automated check is made using the UFRA module.

When one compares the CCL algorithm with other procedures, one sees that for carefully optimized cluster structures a classification purely by energy yields the same result as long as, for example, no enantiomorphic pairs of structures are present in the set of minima. Otherwise, the energy criterion fails, as seen in §3.1.2. Similarly, the CCL algorithm can recognize the similarity of slightly distorted structures occurring, for example, during nonzero-temperature molecular dynamics or Monte Carlo simulations, which exhibit slightly different energies (*cf.* §3.1.4).

Furthermore, the CCL algorithm allows us to address questions like ‘Are two (energetically different) clusters homotops or do they have different underlying atom arrangements?’ by treating all atoms as being of the same type during the cluster comparison. Similarly, the possible rescaling of the clusters makes comparisons between clusters containing different size or type atoms (*e.g.* Zn *versus* Mg) feasible, as seen in §3.1.3.

If one tries to perform cluster comparisons using radial distribution functions, one faces technical issues like the binning of atom distances, but also principal ones like the inability to distinguish enantiomers and the difficulty of comparing clusters consisting of different types of atoms. Analogous problems are encountered by connectivity-based network methods, where the definition of ‘bonds’ is a central parameter and enantiomorphic clusters yield the same network topology. However, as pointed out above, such topological similarities are in some way complementary to the geometric ones addressed by the CCL algorithm.

## Figures and Tables

**Table 1:** Average percentage of how many atom configurations within a randomly selected set drawn from a constant-temperature Monte Carlo simulation are geometrically similar to a given structure from the set

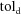	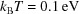 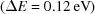 (%)	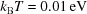 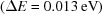 (%)
0.25	0	28
0.5	0.2	87
1.0	7.2	100
1.5	15.5	100

**Table 2:** Number of clusters 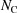, from a set of 3200 minimum Na
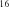
Cl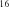 configurations, that contain at least one eight-atom cube (4 Na and 4 Cl atoms) cut out of the bulk rock salt structure

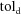	0.1	0.2	0.3	0.4	0.5	0.6	0.7	0.8	0.9	1.0
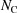	122	722	726	729	740	831	833	834	834	835

## Footnotes

1 Such triplets are relatively efficient, since they allow us to deal with clusters that exhibit a small variation in nearest-neighbor atom distances: since  would be quite sensitive to such differences, possible deviations would propagate when extending the map to the full cluster and thus make it impossible to match the farther-out atoms under the mapping .

2 In order to increase the efficiency and robustness of the algorithm, one should choose as targets atoms of a type of which only a few atoms are present in structure B. If no reference atom type is explicitly selected by the user, CCL employs the atom type with the least number of atoms present in B as a default reference type. Note that in principle it is also possible to assign the same working name to all atoms involved, a situation possibly useful for *e.g.* solid solution alloys.

3 The ten atoms occupy entries 3–12 in the atom list; atoms 1 and 2 in the list are dummy atoms that serve only as reference points.

4
*KPLOT* (Hundt, 2011 ▶) is a standalone program, and can be obtained for free from R. Hundt (hundt@uni-bonn.de) or freely downloaded from the Crystal Impact web site (http://www.crystalimpact.com/download/kplot.htm).
